# A Fruitful Neighborhood

**Published:** 1854-10

**Authors:** 


A Fruitful Neighborhood.—In Wayne County, Pennsylvania, in a circle of seven miles, there live thirteen families, which boast the aggregate number of 195 children.—They are distributed as follows:

| | | | |
|---|---|---|---|
| Jonathan Adams - - - | 18 | Thomas Todd - - - - | 29 |
| Jacob Kellum - - - | 14 | John Phillips - - - - | 12 |
| John Kellum - - - - | 10 | Oliver Bullings - - - - | 13 |
| David Eaton - - - - | 15 | James Brown - - - - | 10 |
| Eben Brown - - - - | 15 | William Tyler - - - - | 10 |
| James Adams - - - - | 14 | Amos Tyler - - - - | 22 |
| Josiah Cole - - - - - | 13 | | |
| Total - - - - - - - - - - - - - - - - - - | | | 195 |

---

The race of mankind would perish, did they cease to aid each other. From the time the mother binds the child's head, till the moment some kind assistant wipes the death damp from the brow of the dying, we cannot exist without mutual help. All, therefore, that need aid, have a right to ask it of their fellow mortals; no one who holds the power of granting can refuse it without guilt.—*Sir Walter Scott.*

---

The opium trade appears to be very brisk at Trenton, N. J. The *Gazette* of that city makes the curious assertion that within two or three years the sale of the drug in the place has increased something like a thousand per cent.

